# The role of PI3Kγ in metabolism and macrophage activation

**DOI:** 10.18632/oncotarget.22068

**Published:** 2017-10-25

**Authors:** Giovanni Solinas, Barbara Becattini

**Affiliations:** Giovanni Solinas: The Wallenberg Laboratory, Department of Molecular and Clinical Medicine, University of Gothenburg, Göteborg, Sweden

**Keywords:** insulin, phosphoinositide 3-kinase, adipose tissue inflammation, tumor-associated-macrophages, neutrophils

**Figure 1:**
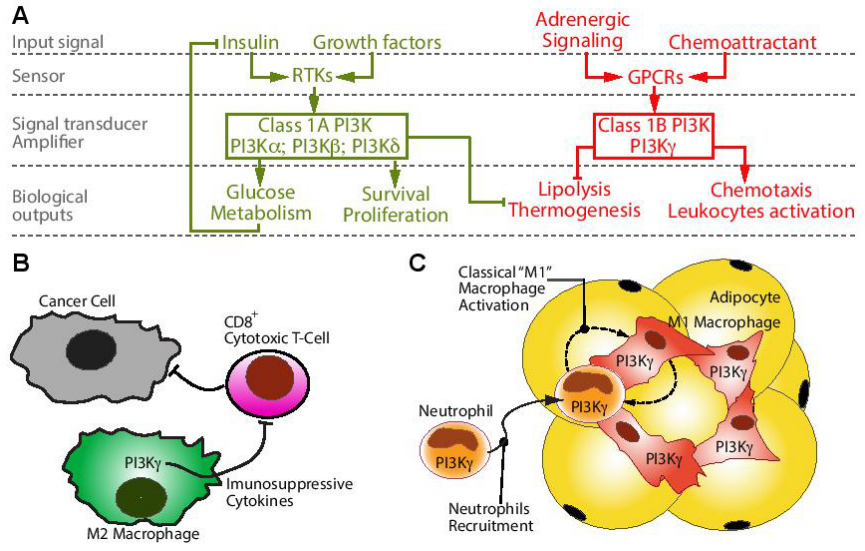
The role of PI3Kγ in metabolism and macrophage activation **A.** Differently from class-1A PI3K signaling (green) the class-1B PI3Kγ (red) was not implicated in growth factor or insulin signaling driven by receptor tyrosine kinases (RTKs), but it is typically recruited by G-protein-coupled receptors (GPCRs) such as β-adrenergic and chemokines receptors. However, it is now appreciated that several cytokines and bacterial byproducts can also activate PI3Kγ. **B.** Recent studies indicate that PI3Kγ activity promotes an M2-activation immunosuppressive phenotype in tumor-associated macrophages, which inhibits CD8^+^ cytotoxic T-cell immune response to different tumor cells implanted in syngeneic mice. **C.** We showed that PI3Kγ activity in leukocytes promotes neutrophil recruitment and classical-M1 inflammatory macrophage polarization in the adipose tissue of obese mice, resulting in an early onset of insulin resistance.

The class 1 phosphoinositide 3-kinase (PI3K) is a family of enzymes which add a phosphate group in position 3 to the membrane lipid phosphatidylinositol-4,5-bisphosphate to generate the second-messenger phosphatidylinositol-3,4,5-trisphosphate. The class-1 PI3K family is further subdivided into class-1A and class-1B. Class-1A PI3Ks comprises PI3Kα; PI3Kβ; and PI3Kδ, which are typically recruited to activated receptor tyrosine kinase such as growth factors receptors and the insulin receptor (Figure 1A). The only class-1B member PI3Kγ is typically associated to G-protein-coupled receptors signaling, including chemokines and β-adrenergic receptors (Figure 1A), although recent studies show that PI3Kγ is also activated by cytokines and bacterial byproducts [1, 2].

Overwhelming evidence consistently indicate that PI3K activity is instrumental to tumor growth and promotion, and PI3K inhibition in cancer therapy is currently one of most intensive area of investigation in biomedical research. However, at present our ability to exploit PI3K as a drug target for cancer therapy is very limited as the results from clinical trials did not meet expectations. It is most likely that dose-limiting side effects and tumor drug resistance are major obstacles which need to be circumvented to exploit the full potential of PI3K as drug target for cancer therapy. Indeed, hyperglycemia and compensatory hyperinsulinemia are common on-target side effects of pan-PI3K inhibitors, and this compensatory hyperinsulinemia is expected to stabilize PI3K activity in hepatocytes and cancer cells (Figure 1A). However, whereas acute PI3K inhibition triggers a transient insulin resistance, over a long term diminished PI3K signaling reduces adiposity and improves insulin sensitivity in mouse and primate models of dietary obesity by a mechanism involving enhanced lipolysis and energy expenditure [1, 3-6] (Figure 1A). Furthermore, we have shown that selective ablation of PI3Kγ in mice does not interfere with insulin-driven glucose metabolism, but reduces diet-induced obesity and insulin resistance [1, 6]. Hence specific PI3Kγ inhibition is not expected to cause compensatory hyperinsulinemia but, at the contrary, it may help to control insulin levels by reducing adiposity in overweight and obese patients.

It should be considered that to date the only PI3K inhibitor used in the clinic for cancer treatment is the PI3Kδ−specific inhibitor idelalisib, which was approved in combination therapies for the treatment of relapsed chronic lymphocytic leukemia (CLL) and other hematological malignancies. Interestingly, idelalisib does not commonly cause hyperglycemia and hyperinsulinemia, and its action on CLL is not due to a cell autonomous activity on the cancer cell but it is mediated by the disruption of signaling loops between the cancer cell and stroma cells. Thus, the success of idelalisib is consistent with the idea that effects on insulin-driven glucose metabolism and on the communication between cancer cells and stromal cells may play a decisive role in the success of specific cancer therapies using isoform-selective PI3K inhibitors.

Recent studies indicate that PI3Kγ promotes an alternative activation (M2) immunosuppressive status in tumor-associated macrophages that prevents CD8^+^ cytotoxic T-cell immune-response to different tumor cell types implanted in syngeneic mice [2, 7]. Thus PI3Kγ-specific inhibitors may be effective in cancer therapies for patients with solid tumors rich in immunosuppressive M2 macrophages, and more specifically in combination therapies with immune checkpoint blocking antibodies [2, 7]. However, these studies are largely based on data from implantation of syngeneic monoclonal tumors in mice and it remains to determine whether PI3Kγ inhibition can promote an effective antitumor immune response in models of chemical carcinogenesis and ultimately in patients.

We have recently investigated the role of PI3Kγ in diet-induced obesity, adipose tissue inflammation, and insulin resistance using different mice models of obesity, and mice with tissue-specific inactivation of PI3Kγ [1, 8]. Mice lacking PI3Kγ are largely protected from diet-induced obesity and insulin resistance, display reduced number of adipose tissue macrophages and reduced expression of markers of classical M1 macrophage activation in adipose tissue [1, 6]. However, in mice with impaired leptin signaling (*ob/ob* and *db/db*) PI3Kγ ablation did not protect from obesity, but delayed the onset of insulin resistance and altered the adipose tissue gene-expression signature indicating that local macrophages were polarized toward alternative “M2” activation [1, 8]. The same phenotype was observed in mice with conditional ablation of PI3Kγ in the endothelial and hematopoietic compartment placed on an obesogenic high-fat diet [1]. Collectively, these results show that PI3Kγ can play opposite actions on macrophage polarization depending on the specific in-vivo context (Figure 1B and 1C). Cultured macrophages lacking PI3Kγ could be efficiently polarized toward M1 activation or M2 activation in-vitro and displayed marginal differences in gene-expression compared to control macrophages [1]. Hence, the in-vivo effects of PI3Kγ on macrophage activation may be mediated by non-cell autonomous mechanisms whose final outcome depends on the specific immune context. Obese mice lacking PI3Kγ in hematopoietic cells showed similar to control amounts of IFNγ; IL-4; and IL-13 in their adipose tissue, indicating that the M2 biased gene-expression profile observed in this tissue may not depend on cell-types controlling the abundance of these cytokines. However, PI3Kγ activity was required for an efficient recruitment of neutrophils to the obese adipose tissue, suggesting a possible crosstalk between adipose tissue neutrophils and macrophages supporting M1 proinflammatory gene-expression in this tissue [1, 8] (Figure 1C).

We conclude that whereas PI3Kγ can play an important role in macrophage gene expression, this action is not cell autonomous and depends on PI3Kγ activity in different cell types, including neutrophils. For the future, it will be important to investigate the role of PI3Kγ in different models of chemical carcinogenesis and in patients with different types of solid tumors as the effects of PI3Kγ inhibition on the tumor microenvironment may depend on the specific context. It remains also to determine whether PI3Kγ activity is required for the recruitment of tumor-associated neutrophils and granulocytic myeloid-derived suppressor cells.
